# Author Index for Volume 94

**DOI:** 10.1038/sj.bjc.6603217

**Published:** 2006-06-13

Aaboe, M 1465

Aaltonen, K 1697

Abecassis, J 1041

Achen, MG 1355

Ackers, C 1231

Adam, E 1734

Adighibe, O 1176

Agus, DB 85

A'Hern, RP 631

Ahlin, C 1697

Ahmad, T 74

Ahmed, AA 1326

Ahmed, S 1921

Aitchison, M 781

Aithal, GP 1751

Ajioka, Y 293

Akazawa, K 1510

Akino, K 914

Akiya, T 1803

Al Murri, AM 227

Alam-Fahmy, M 1906

Al-Batran, S-E 1615

Albini, A 1428

Alfsen, GC 820

Alken, P 578

Allan, S 828

Allen, NE 299

Allgood, PC 36

Alliot, C 935, 938

Altundag, K 338

Amini, R-M 1697

Ammerpohl, O 323

Andersen, JB 1465

Anderson, E 1021

Anderson, JH 1833

Anderson, KE 757

Anderson, TJ 1051, 1333

Andersson, S 1045

André, T 1287

Androulakis, N 798

Ang, R 346

Angerson, WJ 1833

Ångstrom, T 1913

Ansieau, S 13

Anthoney, DA 1281

Antoine, M 473

Antonescu, C 1770

Antonopoulos, M 947

Anttinen, J 147

Aprile, G 785

Ardanaz, E 299

Ardern-Jones, A 308

Armer, T 1021

Arnould, L 259

Aromaa, A 1245

Artru, P 1287

Arumuguma, M 1204

Ashley, S 18, 43, 358

Athanasiadis, A 798

Athanasou, NA 1496

Atkin, G 121

Atkinson, R 62

Atmaca, A 1615

Atzpodien, J 1785

Auborn, K 407

Auvinen, A 1352

Åvall-Lundqvist, E 1683

Avallone, A 1809

Aversa, SML 1550

Avis, M 1253

Baas, P 625

Baba, K 1599

Babiker, A 1504

Bachelot, T 1066

Bachman, KE 455

Bachmann, MO 36

Backe, J-C 1342

Bae, D-S 1678

Bae, J-M 1402

Baek, JH 1407

Baeuerle, PA 128

Bagley, S 842

Bagstaff, SM 1936

Bai, J 686

Baildam, AD 1021

Balañá, C 1797

Baldini, E 1263

Baldus, C 43

Bali, A 904

Balinandi, S 446

Ballestrero, A 1016

Baltaci, M 835

Balzano, G 785

Bamford, S 318

Banna, GL 1550

Baptista, MZ 338

Baptiste, MS 1738

Baranowski, AP 178

Baras, A 1436

Barbera, C 743

Barget, N 287

Barnes, N 253

Baron-Hay, S 904

Barr, H 1460

Barraclough, R 1057

Barranco, WT 884

Bartlett, JB 1412

Bartlett, JMS 227

Basten, GP 1942

Batra, RK 1029

Beale, P 964

Beale, PJ 208

Beaugrand, M 287

Beaune, P 692

Beckmann, JS 1537

Beham, A 1122

Beijnen, JH 1226

Belderbos, JSA 625

Bell, RF 1559

Bellec, S 763

Benamouzig, R 1823

Ben-Asher, E 1537

Bengala, C 1016

Bennett, G 593

Bennouna, J 69, 1383

Benoit, L 259

Benoy, IH 672

Beomonte Zobel, B 792

Beral, V 1504

Bergenheim, AT 1853

Berger, F 1066

Berkhof, J 171

Berking, C 1879

Bernardini, R 1428

Bernaudin, J-F 1164

Berrino, F 299

Bertetto, O 1122

Bertheau, P 259

Berthois, Y 239

Beskow, C 1683

Bessaguet, C 1214

Betticher, DC 1099

Bhopal, R 1079

Bianco, AR 1604

Bicknell, R 1

Biessy, C 299

Billingham, LJ 287, 1898

Bingham, SA 299

Bingle, L 101

Birch-Machin, MA 1887

Birembaut, P 724

Biryahwaho, B 446

Bischof Delaloye, A 1770

Bischoff, J 1615

Bishop, H 828

Black, J 1107

Blay, J-Y 1066

Blohmer, J-U 1237

Blomqvist, C 1697

Blons, H 692

Bloomfield, D 828

Bogdanova, T 1472

Bohle, RM 323

Boillot, C 299

Bojesen, SE 323

Bolaños, M 969

Bomsztyk, K 586

Boneberg, E-M 524

Bonetto, E 785

Bonin, S 879

Bonnetain, F 259

Borden, EC 1465

Bordenave, S 69

Borgoño, CA 540

Borkhardt, A 323

Boshoff, C 1504

Botti, G 1233

Bouchardy, C 231

Bouchier-Hayes, DJ 1320

Bourboulia, D 1504

Bourne, S 121

Bouvet, M 1311

Boven, E 1627

Box, C 631

Boyer, MJ 208

Brabletz, T 1672

Brągoszewski, P 586

Brain, E 1610

Bramley, M 1021

Brandes, AA 785

Bras, J 1837

Braselmann, H 1472

Brasnu, D 692

Breau, J-L 1823

Bregni, M 1016

Breitbach, G-P 1237

Brenner, H 450

Brenton, JD 1326

Breton, J-L 1383

Bria, E 1789

Bricaire, F 1000

Brock, A 1079

Brock, C 1217

Brodie, AMH 513

Brooks, CL 1555

Brophy, S 1320

Brougham, B 43

Brouty-Boyé, D 1180

Brown, G 351

Brown, JE 469

Brown, JS 561, 647

Brown, KA 1144

Brown, MD 842

Brown, MM 1621

Brown, NJ 101

Brown, P 1650

Brown, R 1087

Brown, SC 1621

Brownbill, PA 22

Broxterman, HJ 1627

Brunner, S 1726

Bryzgunova, OE 1492

Bubendorf, L 128

Buchegger, F 1770

Budczies, J 1672

Budillon, A 1809

Budner, M 1237

Bueno-de-Mesquita, HB 299

Buesa, JM 1797

Bui, B 1395

Bujnicki, JM 586

Bukawa, H 698, 717

Bulk, S 171

Bulkmans, NWJ 171

Bulten, J 814

Bundred, NJ 253

Bunstead, Z 507

Buonaguro, FM 446

Buonaguro, L 446

Burge, ME 1281

Burgers, JA 625

Buring, JE 757

Burkhardt, M 540

Burton, C 806

Burton, S 351

Burtt, G 569

Bustová, I 1122

Cabaret, V 259

Cahill, S 398

Caldas, C 1326

Caldwell, GM 922

Callard, P 1164

Campbell, NC 1272

Campo, L 1176

Canney, PA 227

Cantarella, G 1428

Canzian, F 299

Caporale, R 1637

Caputo, F 1604

Cardenal, F 1375

Cardis, E 1352

Cardoso, T 1621

Carlini, B 1845

Carlini, P 1789

Carpenter, L 1504

Carreon, JD 1690

Carter, T 407

Casabonne, D 1504

Casado, A 1797

Casado, E 969

Cascini, LG 1809

Casparie, MK 752

Cassidy, J 1122

Castano, AP 391

Catalano, G 1604

Cataldo, D 724

Caumes, E 1000

Cecere, FL 1789

Cellai, C 1637

Cerny, T 1099

Cerundolo, V 1496

Chae, YS 1407

Chaganti, RSK 614

Chajès, V 299

Chakraborty, C 1154

Chamorey, E 1516

Chan, AT 928

Chan, JK 642

Chan, S 55

Chandanos, E 136

Chang, JT 870

Chang-Claude, J 299

Chao, D 1217

Chappuis, PO 231

Chari, R 1927

Charnock, MFL 1650

Chartier, M 1342

Chastang, J-F 152

Chatrath, P 1170

Chaturvedi, A 427, 1554

Chau, I 1107

Chechlińska, M 1761

Chen, D 1555

Chen, E 1136

Chen, L 642

Chen, Y-J 870

Chen, Y-L 870

Cheng, A-J 870

Cheng, Y 287, 1898

Cheok, MH 93

Cheung, MK 642

Chiarion-Sileni, V 1550

Chiarugi, A 743

Chiew, KS 1011

Chiodini, P 1233

Chirlaque, M-D 299

Cho, GJ 1407

Cho, Y 275

Choi, CH 1678

Choi, IJ 1402

Choi, J-J 1678

Choi, YJ 1407

Chokunonga, E 450

Chong, G 937

Chowdhry, S 1913

Chua, A 1436

Chun, JH 1402

Chung, JS 1407

Cianfrocca, ME 1621

Ciardiello, F 1604

Ciccarese, M 1789

Cinti, R 1845

Clamp, AR 55

Clarke, NW 842

Clarke, RB 1021

Clarke, SJ 208, 964

Clavel, J 763, 1342

Clavel-Chapelon, F 299

Clements, J 318

Cleton-Jansen, A-M 661

Cleveland, RJ 299

Coe, BP 1927

Coebergh, JWW 752

Cognetti, F 1548, 1789

Cohen, M 79

Cohen, RB 1621

Colantuoni, G 1604

Colditz, GA 928

Coleman, N 1170

Coleman, R 62

Coleman, RE 30, 469

Colin, C 1383

Colpaert, CG 1643

Colquitt, JL 982

Comella, P 1809

Conan-Charlet, V 1214

Conte, PF 1016, 1263

Copeland, M 308

Coppola, R 792

Cordio, S 785

Corke, KP 101

Cornelisse, CJ 661

Corpuz, MR 654

Corrias, MV 1845

Cotton, SC 1253

Coudert, B 259

Coulet, F 473

Coulson, P 499

Coursaget, P 1949

Cowan-Jacob, S 1765

Coward, J 18

Cowell, W 1122

Coxon, F 1122

Coy, JF 578

Craft, AW 22

Craig, M 1777

Crivellari, G 1550

Crozier, JEM 1833

Cruickshank, D 55

Cruickshank, M 1253

Crumley, ABC 637, 1568

Cucherat, M 1823

Culine, S 1395

Cunningham, D 351, 806, 937, 1107

Cunningham, MS 189

Cutz, J-C 1452

Cuzick, J 460

Cvitkovic, F 69

Czarnecki, A 1759

Da Prada, G 1016

Dahl, E 540

Dahl, K 1478

D'Aiuto, G 1233

Dale, J 372

Daley, FM 121

Dalgleish, AG 1412

D'Ambrosio, C 631

Dancey, J 1136

Daniels, F 1107

Daniels, IR 351

Danova, M 1016

Darbyshire, J 1504

Davidson, SE 115

Davies, HA 469

Davies, MPA 1057

Davies, RJ 1170

Dawson, E 318

De Castro, J 969

de Gramont, A 1287

de Hullu, JA 814

de Jonge, ME 1226

de Koning, HJ 1093

De Laurentiis, M 1604

De Maio, E 1233

de Matteis, A 1233

de Mon, Má 969

De Placido, S 1604

De Santis, M 1395

de Silva Rudland, S 1057

De Vita, F 1604

Deans, DAC 731

Dearnaley, D 499, 507, 1361

Deasy, JM 1320

Debus, J 1375

Del Freo, A 1263

Deleuze, J 1000

Delgado, F-M 1395

Dell'Eva, R 1428

Dell'Oro, S 785

Delrio, P 1809

Demkow, T 1395

Demols, A 481

Dempsey, GA 647

Denisenko, O 586

Denkert, C 540

Des Guetz, G 1823

Desruisseau, S 239

Dessypris, N 156

Deutsch, G 828

Di Carlo, V 785

Di Cosimo, S 1789

Di Lollo, S 1637

Di Maio, M 1233

Diadema, MR 1604

Diamandis, EP 540

Díaz-Rubio, E 1122

Dierssen, JWF 661

Dietel, M 540

Dietl, B 1544

Dikalioti, SK 156

Dillner, J 1913

Dirix, LY 672, 1643

Dittmer, J 176

Ditzel, HJ 1864

Dixon, JM 1051, 1333

Dixon, S 492

Djuzenova, CS 1194

Doak, SH 891

Dodd, M 647

Dodwell, D 30

Dogan, A 318

Doi, T 1803

Doihara, H 247

Donskov, F 218

Doran, P 1204

Dorval, E 69

Dossus, L 299

Doughty, JC 227

Douillard, J-Y 69, 1122, 1383

Downing, R 446

Doyle, E 1204

du Bois, A 79

du Manoir, S 1041

du Plessis, DG 1186

Dubinett, SM 1029

Dufour, P 1122

Dugas, B 854

Dunn, JR 1186

Dunning, AM 1921

Dupin, N 1000

Duraturo, ML 446

Duthie, SJ 1942

Duyndam, MCA 1627

Dyba, T 1245

Dyer, MJS 806

Dzwonek, A 586

Easton, DF 1921

Ebi, KL 161, 940

Eccles, SA 631

Eccleston, C 1559

Eckhert, CD 884

Edwards, DR 569

Edwards, L 43

Eeles, R 308, 499, 507

Egli, F 1099

Eickhoff, A 1572

Eickhoff, JC 1572

Eijkenboom, WMH 1389

Eilers, PH 661

Eisen, T 1217

Eisenberg, I 681

Eisendle, K 835

Eiser, C 469

Eisinger, F 340

Ekman, P 1658

Elling, D 1237

Ellis, LM 1710

Elst, H 672

Emile, J-F 1180

Emlet, DR 1144

Endo, Y 698, 717

English, WR 1326

Era, S 1874

Erlach, N 1718

Erlandsson, F 1045

Esaki, T 1803

Escudero, P 969

Essink-Bot, M-L 1093

Esteban, E 1797

Esteller, M 179

Esterman, A 1116

Evans, C 1412

Evans, DGR 1021

Evans, WE 93

Evrard, A-S 1342

Ewertz, M 1339

Fabbri, A 1263

Fabbrocini, G 743

Fabi, A 1789

Falcone, A 1263

Fan, F 1710

Fan, S 407

Fan, Y 1658

Fang, C-W 870

Fang, X 1658

Fearon, KCH 731

Fehn, M 1194

Felici, A 1548, 1789

Feliu, J 969

Ferguson, M 1176

Fermeaux, V 259

Ferretti, G 1548, 1789

Feychting, M 1352

Field, JK 561

Fietkau, R 976

Figer, A 1122

Fink, D 904

Fink, K 268

Finlayson, C 1412

Fioretta, G 231

Fischbacher, CM 1079

Fjällskog, M-L 1697

Flanagan, A 318

Flentje, M 1194

Fles, R 333

Flesch, M 1287

Flint, P 253

Foidart, J-M 724

Foitzik, T 976

Fokdal, L 1703

Foliart, DE 161, 940

Fontijn, D 1627

Forbes, S 318

Formento, J-L 1516

Fosså, SD 820

Fountzilas, G 1122

Fox, SB 1

Fra, J 1797

Franke, A 200

Frankendal, B 1683

Freudenheim, JL 757

Freund, HR 681

Frickhofen, N 79

Friedman, E 1537

Friis, S 1339

Fritsch, PO 835

Fritz, GI 1726

Fritzsche, F 540

Fuchs, CS 928

Fuchs, U 323

Fugazza, C 785

Fujita, T 247

Fujita, Y 737

Fujiwara, M 1485

Fukuda, M 1267, 1267

Fulton-Kehoe, D 1071

Furrer, M 1099

Fürstenberger, G 524

Futreal, PA 318

Gafà, L 743

Gal, I 1537

Galán, A 969

Galica, J 1011

Galle, PR 1572

Gallick, GE 1710

Gallo, C 1233

Gallo, J 1621

Gambotti, L 1000

Ganesan, TS 1650

Gansukh, T 540

García del Muro, J 1797

García Rodríguez, LA 136

Garden, OJ 213, 982

Gardiner-Garden, M 904

Garnier, J 259

Garrison, LP 1122

Garte, S 1533

Gatter, K 1176

Gay, F 481

Gazdar, AF 1927

Gazi, E 842

Gee, AL 1186

Gelibter, A 1789

Gelly, M 259

Genestie, C 473

Genkinger, JM 757

Georgoulias, V 798

Gerritsen, WR 1837

Ghali, L 1446

Ghiotto, C 1550

Giannarelli, D 1789

Gibault, L 1214

Gibbens, I 74

Giele, H 1496

Gillbanks, A 1107

Gilliland, G 1918

Gilmour, H 731

Giovannucci, E 928

Girard, L 1927

Gislum, M 142

Giusti, C 239

Gjerstorff, MF 1864

Glasspool, RM 1087

Glynne-Jones, R 121, 363

Goedert, JJ 548

Goekkurt, E 281

Going, JJ 1568

Goldbohm, RA 165, 757

Goloubeva, OG 1465

Gondos, A 450

González-Barón, M 969

Gook, D 1007

Gore, M 74, 1217

Gorin, I 1000

Goubin, A 763

Graat, HCA 1837

Graeven, U 1293

Grainger, J 363

Gravina, A 1233

Gray, NM 1253

Green, J 1446

Greenfield, DM 469

Gregersen, H 1339

Greinert, R 743

Gridelli, C 1604

Grieu, F 593

Griffin, JD 1765

Griffioen, G 1035

Griffiths, C 1079

Griffoni, C 1300

Grigg, A 1007

Grobholz, R 578

Grusch, M 1718

Grzesiak, JJ 1311

Gu, W 1555

Guan, XY 108

Guarneri, V 1016

Gueders, M 724

Guida, C 1809

Haas, OA 323

Habeshaw, T 74

Habicht, J 1099

Hacker, NF 904

Hackman, J 1777

Hägg, M 1592

Hallmans, G 1913

Halstead, F 1281

Hamacher, J 524

Hamblin, MR 391

Hamilton, CA 642

Hammett, Z 1116

Han, C 1650

Hanagiri, T 896

Hancock, B 806

Hancock, BW 469, 492

Hanemaaijer, R 1035

Hankinson, SE 757

Hanly, AM 1320

Hannoun-Lévi, J-M 1516

Hansen, E 1099

Hansson Mild, K 1348

Harbottle, A 1887

Hardell, L 1348

Harnack, L 757

Harper, P 1107

Harries, M 1217

Harrington, KJ 631

Harris, A 1176

Harrison, M 363

Harrison, P 1107

Hart, CA 842

Hartmann, D 1572

Hasegawa, Y 1599

Hashimoto, M 1894

Hassel, BA 1465

Hassler, E 835

Hasson, P 771

Hatch, EE 1734

Hauser, P 1918

Hayes, DF 8

Haywood, P 253

Hebbar, M 69

Hecker, D 1615

Hedley, D 1136

Heikkilä, P 1697

Heinzelmann-Schwarz, VA 904

Helder, MN 1837

Helg, C 1770

Hellström, A-C 1045

Hémon, D 763, 1342

Hendlisz, A 481

Hendrickson, MR 642

Hennig, E 586

Henriksson, R 1853

Henshall, SM 904

Herbarth, O 1726

Herbst, A 1734

Herings, RMC 752

Heriot, AG 1412

Hewitt, SM 686

Hickish, T 1107

Hicklin, DJ 1710

Hieber, L 1472

Higashi, H 1874

Higginson, A 1420

Higo, M 717

Hill, MH 1942

Hillinger, S 1029

Hindley, A 55

Hiraoka, K 275

Hirayama, Y 311

Hiyama, E 1510

Hlubek, F 1672

Ho, J 1136

Hocking, B 1350

Hodson, N 828

Hoehn, S 281

Hofstetter, W 1496

Hogervorst, FBL 333

Hogue, CJR 1745

Hokland, M 218

Holgersson, Å 1683

Hollywood, D 189

Holzmann, K 1718

Honegger, H 1099

Hoogerbrugge, N 814

Hoover, RN 1734

Hopwood, P 507

Horgan, PG 1833

Horsfield, MA 1420

Horsman, JM 30

Horvath, L 964

Hoskin, P 806

Hosoi, H 1510

Hosokawa, M 854

Hossfeld, DK 281

Houben, MPWA 752

Houllier, AM 692

Howell, A 460, 1021

Hsu Schmitz, S-F 1099

Huat, TE 1383

Hubbard, A 427, 1554

Hubner, R 74

Hudes, G 1621

Hudson, TJ 436

Huitema, ADR 1226

Hull, R 1107

Hung, M-C 184

Hunter, DJ 757, 928

Hunter, RD 115

Hussenet, T 1041

Hutchison, C 486

Hutzler, P 1472

Huynh, CK 513

Hwang, H-W 776

Hyer, M 1734

Hyodo, I 1803

Iacopetta, B 593

Iacopetta, BJ 339

Ichikawa, S 737

Ichikawa, W 1130

Ichiki, Y 896

Iehara, T 1510

Ijichi, T 854

Ikemura, K 1580

Ikram, M 1446

Imai, K 914

Imaizumi, K 1599

Imawari, M 311

Inenaga, R 1580

Innes, HE 1057

Innocenti, F 1263

Ino, K 552

Inokuchi, M 1130

Inoue, H 1894

Inoue, M 740

Iriye, R 161, 940

Ishida, E 532

Ishihara, T 1575

Ishikawa, K 1894

Ishikawa, Y 1485

Ismail, T 1898

Ito, H 311

Itoh, T 275

Ittah, H 1000

Iuchi, Y 854

Ivil, KD 891

Iwai, N 737

Iwasaki, M 740

Iyer, NG 1326

Izumi, H 710

Izutsu, T 1586

Jack, A 806

Jackson, D 1650

Jackson, DP 1281

Jacob, JH 69

Jager, D 681

Jager, E 1615

Jain, A 85

Jakobs, R 1572

James, N 1395

Janssens, ACJW 1093

Jayson, GC 55

Jeannin, J-F 259

Jelic, S 1122

Jenkins, SA 891

Jenkins, V 828

Jentsch-Ullrich, K 200

Jeon, SB 1407

Jeremiah, S 1472

Jimeno, J 1610

Johansen, C 1352

Johansen, LE 1864

Johansson, M 1853

Johns, L 499

Johnsen, HE 1339

Johnson, H 1898

Johnson, PJ 287, 1898

Johnston, PG 1122

Jones, CE 922

Jones, RL 358

Joosse, SA 333

Jordan, C 1921

Joss, C 1099

Jovic, G 1016

Jung, A 1672

Jung, B 79

Jung, HY 1407

Jung, K 540

Kaaks, R 299

Kabuubi, P 115

Kajiyama, H 552

Kakolyris, S 798

Kalindjian, B 1107

Kalso, E 1559

Kalykaki, A 798

Kan, C-Y 339

Kanazawa, T 293

Kaneko, K 311

Kaneko, M 1510

Kanter, L 1683

Kapp, DS 642

Karakas, B 455

Karczmarski, J 586

Karlsson, M 1478

Kasamatsu, A 717

Kaspers, GJL 1837

Katlama, C 1000

Kato, H 1575

Kato, K 1599

Kato, T 1803

Katoh, H 275

Kaufman, R 1734

Kaur, K 1650

Kautiainen, H 147

Kavet, R 161, 940

Kawakami, K 593

Kawano, T 1130

Kawasaki, K 247

Kay, EW 1320

Kaye, S 62, 74

Kazama, S 293

Kehoe, S 1

Kelesidis, I 1221

Kelesidis, T 1221

Kellokumpu-Lehtinen, P 1245

Kelly, JD 569

Kendall, C 1460

Ketterer, N 1770

Key, TJ 299

Keyzor, C 74

Khan, AN 1213

Khaw, K-T 299

Kheifets, L 161, 940

Kigawa, J 1369

Kikkawa, F 552

Kikuchi, Y 1369

Killing, B 1293

Kim, B-G 1678

Kim, B-S 959

Kim, CG 1402

Kim, DH 1407

Kim, HK 1402

Kim, JG 1407

Kim, RH 620

Kim, T-J 1678

Kim, YW 1402

Kimmel, G 1537

Kimmel, KA 1621

Kinjo, F 1803

Kinoshita, A 1267

Kirby, AM 631

Kirchner, T 1672

Klar, E 976

Klautke, G 976

Kleeberg, U 1615

Kleifeld, O 941

Klimek-Tomczak, K 586

Knox, WF 253

Knuth, A 1615

Kobayashi, M 854

Kobayashi, T 854

Kobylecki, C 115

Kock, K 1864

Koda, K 1816

Koenigsmann, M 200

Kofler, B 268

Kohls, A 1237

Kohno, K 710

Kohno, S 1267

Kohno, Y 710

Koike, H 717

Koizumi, W 1803

Kojima, K 1130

Kölbl, O 1544

Komatsu, Y 1803

Kondo, S 275

Konishi, K 311

Konishi, N 532

Kononen, J 128

Koren, M 1537

Korfage, IJ 1093

Kosinski, M 1770

Kosmidis, P 947, 1383

Kote-Jarai, Z 308

Koumakpayi, IH 1906

Kouroussis, Ch 798

Kouzu, Y 717

Kovacsovics, T 1770

Kowalewska, M 1761

Kramer, G 1592

Kremer, B 1293

Kristiansen, G 540

Krocker, J 1237

Kroll, ME 22

Krubasik, D 1326

Kuba, M 1267

Kubben, FJGM 1035

Küchenmeister, U 976

Kumar, D 1412

Kumekawa, Y 311

Kümmel, S 1237

Kuopio, T 147

Kurachi, H 1586

Kurahashi, N 740

Kurahashi, T 311

Kurozawa, Y 737

Kurschat, P 1086

Kurzawinski, T 1107

Kusafuka, T 1510

Kusano, M 311

Kuznetsova, NP 1492

Kuzuya, K 1369

Kwa, HB 625

Kwan, A 1504

Kynaston, HG 891

Labonia, V 1233

Lacau St Guily, J 1164

Lacave, R 473, 1164

Laccourreye, O 692

Lade, S 1007

Lagarde, N 1214

Lagergren, J 136

Lagos, D 1504

Lahmann, PH 299

Lahousen, M 947

Lai, C 1553

Lakhani, SR 358

Laktionov, PP 1492

Lala, PK 1154

Lam, GN 1621

Lam, S 1927

Lam, WL 1927

Lamb, GWA 781

Lamers, CBHW 1035

Lamph, WW 654

Lancet, D 1537

Landi, B 1287

Landi, G 1233

Landi, S 299

Langagergaard, V 142

Langbein, S 578

Larrañaga, N 299

Larsson, P 1478

Larsson, SC 757

Lash, TL 142

Laskey, RA 1170

Lastoria, S 1809

Lau, D 1452

Lau, YS 1496

Laurent-Puig, P 692

Laurenzana, A 1637

Laurier, D 1342

Laux, I 85

Lawson, JS 339

Le Bacquer, O 195

Le Page, C 436, 1906

Lebeau, B 1375

Leclerc, A 152

Ledermann, J 55

Lee, CM 863

Lee, J-H 1402, 1678

Lee, JS 1402

Lee, J-W 1678

Lee, KB 1407

Lefèvre, M 1164

Lefranc, JP 473

Lehto, U-S 1245

Leigh, IM 1446

Leitzmann, M 757

Lemoine, A 1180

Lempereur, L 1428

Lerbs, W 1615

Lesniewski-Kmak, K 1122

Lesslie III, DP 1710

Lester, JE 30

Lesueur, F 1921

Leung, HY 569

Lewensohn, R 1683

Lewis, CE 101

Lewis, N 1621

Lewsley, L 62

Leyden, J 1204

Li, XD 1913

Lièvre, A 692

Ligtenberg, MJ 333, 814

Liloglou, T 561

Linch, D 806

Lind, M 947

Lind, MJ 427, 1554

Lindblad, M 136

Linder, S 1592

Link, MP 161, 940

Linseisen, J 299

Little, J 1253

Liu, D 1057

Liu, N 1452

Liu, Q 391

Liu, S 686

Liu, Z 1658

Lledo, G 1287

Lo, H-W 184

Locke, I 308

Lockwood, WW 1927

Loehr, A 79

Logan, RFA 1751

Lombaerts, M 661

Loncaster, JA 115

Longacre, TA 642

Longerey, B 1395

Lopez-Gómez, L 969

López-Pousa, A 1797

Lorenz, J 1395

Loria, DI 743

Losa, F 969

Losa, R 1797

Lotz, JP 473

Lou, F 1658

Louvet, C 1287

Lowe, D 561, 647

Lowrie, AG 1663

Lowry, M 427, 1554

Lozac'H, P 1214

Lu, Y-C 870

Lu, Y-J 308

Luben, R 1921

Lucas, R 524

Lucas, SB 446

Luccarini, C 1921

Luce, D 152

Ludwig, K 976

Lukan, N 578

Luppi, G 785

Lutze, G 200

Ma, J 928

Mabro, M 1287

MacAulay, C 1927

Macdonald, S 1272

Mach, J-P 1770

MacLennan, K 806

Macleod, U 1272

MacMathuna, P 1204

Madhavan, KK 213

Madore, J 436

Maeda, Y 1803

Mäenpää, J 55

Maindrault-Goebel, F 1287

Major, P 1136

Mak, TW 620

Makino, R 311

Makris, A 363

Malek, K 69

Mall, M 1472

Malzyner, A 1122

Mamane, Y 195

Manara, MC 1300

Mandelson, MT 1071

Mandic Havelka, A 1592

Manegold, C 1375

Manek, S 1650

Manley, P 1765

Mann, GB 1355

Manton, DJ 427, 1554

Mantzoros, CS 156, 1221

Maraveyas, A 427, 1554

Marcussen, N 218

Marechal, R 481

Marian, B 1718

Marits, P 1478

Markmann, I 200

Markowicz, S 1761

Marone, P 1809

Marshall, J 757

Martin, A 287, 1898

Martin, C 955

Martín, J 1797

Martin, P-M 239

Martin, W 1572

Martinez, C 743

Martinez, V 1000

Martínez-García, C 299

Martínez-Trufero, J 1797

Martino, M 1016

Martinsson-Ahlzén, H-S 1045

Maruyama, R 914

Mascaux, C 1549

Mason, B 351

Masson, LF 1253

Massuger, LF 814

Matakidou, A 18

Matsubara, N 854

Matsuki, Y 710

Matsumoto, S 1599

Matsumoto, Y 1510

Matsuyoshi, S 532

Matteucci, P 1016

Matthews, GM 922

Mattson, K 1375

Mauch, C 1086

Maughan, TS 1122

Maur, M 1016

Maurel, J 1797

Mauritz, I 1718

Mavroudis, D 798

Maxhimer, JB 1436

May, J 1777

Mayordomo, E 540

Mayr, JA 268

Mazar, AP 1621

Mazhar, D 346

McArdle, CS 1833

McCullough, ML 757

McDonald, AC 637

McEntee, G 1204

McGorm, K 1116

McKay, JD 299

McKee, RF 1833

McKendrick, J 1007

McKendrick, JJ 1122

McKernan, M 637, 1568

McKinney, PA 1352

McLaughlin, CC 1738

McLinton, A 486

McMillan, DC 227, 637, 781, 1568, 1833

Meazza, R 1845

Meierhofer, D 268

Meijer, CJLM 171

Meldgaard, P 1703

Melia, J 499, 507, 1361

Memon, AA 1703

Mendell, JT 776

Menetrier-Caux, C 1066

Meng, Q 407

Mengitsu, T 308

Menvielle, G 152

Mercier, M 743

Mes-Masson, A-M 436, 1906

Mestan, J 1765

Mestel, DS 1879

Metcalf III, CA 1710

Metges, J-P 1214

Mettal, H 323

Meuthen, I 1615

Meyer, M 398

Mezei, G 161, 940

Micklem, K 1176

Middleton, G 1107

Miertusova Tothova, S 879

Migeon, C 259

Mikula, M 586

Milandri, C 785

Milella, M 1789

Milham, S 1351, 1354

Miller, AB 757

Miller, WR 1051, 1333

Milligan, D 806

Millon, R 1041

Mills, IG 569

Mimori, K 1894

Minna, JD 1927

Miranda, A 743

Misset, JL 1610

Mitchell, E 1272

Mitchell, P 1007

Mitra, S 828

Mitrani-Rosenbaum, S 681, 1762

Miyamoto, M 275

Miyata, Y 1803

Miyazaki, M 1816

Mizunuma, H 1586

Möhler, M 1572

Mohren, M 200

Mommers, M 165

Monfardini, S 1550

Monnier, S 299

Monsaert, E 481

Moore, J 593

Moore, MJ 1136

Morabito, A 1233

Morack, G 1237, 1615

Morere, J-F 1823

Morgan, B 1420

Mori, M 1894

Mori, S 30

Morin, A 1342

Morini, J-P 1000

Morita, M 896

Moriya, T 1369, 1586

Morrica, B 1809

Morris, LS 1170

Morris, R 828

Morrison, I 1412

Morton, DG 922

Moss, A 1204

Moss, S 499, 507, 1361

Mossotti, R 743

Motzer, RJ 614

Mouncey, P 806

Moureau-Zabotto, L 473

Moynihan, C 499, 507

Mueller, BU 1918

Mühl, B 1194

Müller, B 1194

Muller, D 1041

Muller, K 1389

Munakata, M 1803

Munaut, C 724

Munoz, F 1504

Murphy, G 569, 1326

Murray, D 1204

Murray, G 1107

Murray, J 1051

Muston, D 1361

Nagasaka, T 552

Nagashima, S 1267

Nagawa, H 293

Nakagawa, K 1485

Nakahara, K 1586

Nakakubo, Y 275

Nakamura, K 1575

Nakamura, M 532

Nakamura, S 1580

Nakamura, Y 1267

Nakano, S 1803

Nakashima, D 717

Nakata, S 896

Nakayama, K 311

Nanni Costa, A 1533

Narahara, H 1803

Nasca, PC 1738

Nathan, P 1217

Navarro, C 743

Nawa, A 552

Neal, DE 569

Nederlof, PM 333

Negro-Vilar, A 654

Neuberg, D 1918

Nevanlinna, H 1697

Newton, R 1504, 1949

Nexo, E 1703

Nguyen, DM 1436

Nicklee, T 1136

Nico, B 1845

Nicolas, P 1823

Nicoletti, R 785

Nielsen, O 1864

Nieto, A 743

Nihei, Z 1130

Nikitenko, LL 1

Nilsson, B 1683

Nilsson, S 1658

Ninomiya, H 1485

Nishikawa, N 914

Nishimura, M 1816

Nishizaki, T 1874

Nissan, A 681, 1762

Njar, VCO 513

N'Kontchou, G 287

Noel, A 724

Nomura, K 1485

Nomura, S 552

Noonan, DN 1428

Norat, T 299

Nørgaard, M 1339

Nørgård, B 142

Norman, A 507

Norman, AR 351, 1107

Normanno, N 1233

Northover, J 121

Norton, A 18

Nose, T 737

Nowak, R 1761

Nozawa, H 311

Nutting, CM 631

Nuzzo, C 1789

Nuzzo, F 1233

O'Brien, M 1383

O'Brien, MER 18

O'Brien, PM 904

Oda, K 1816

Odell, E 1170

O'Donnell, J 189

O'Dwyer, M 398

Oei, AL 814

Ogasawara, Y 247

Ogi, K 914

Ogimoto, I 737

Ohbuchi, T 275

Ohyashiki, JH 599

Ohyashiki, K 599

Oishi, T 1599

Ojanen, M 1245

Oka, M 1267

Okada, F 854

Okamura, K 1586

O'Keane, C 1204

Okumura, S 1485

Old, LJ 681

Oldenburg, J 820

Onda, T 698

O'Neill, PA 1057

Onesto, C 1516

Ong, S 964

Ono, K 896

Onuma, K 854

Oosting, J 661

Oppitz, U 1194

O'Quigley, J 609

Orditura, M 1604

Ørntoft, TF 1465

Osann, K 642

Osborne, R 62

Oshikiri, T 275

Østerlind, A 743

Ostler, P 363

Ostrowski, J 586

Ota, H 311

Otani, T 740

O'Toole, EA 1446

O'Toole, L 469

Ottensmeier, C 1383

Ouellet, V 436

Overall, CM 941

Oya, R 1580

Oyama, T 896

Oza, AM 1136

Pabst, T 1918

Pacilio, C 1233

Pagès, G 1516

Paglierani, M 1637

Pahl, S 540

Paillot, B 69

Palli, D 299

Palmari, J 239

Palmer, JR 1734

Palmieri, G 1604

Pancrazzi, A 1637

Panetta, JC 93

Paoletti, F 1637

Papa, MZ 1537

Papaldo, P 1789

Pappin, C 51

Paracchini, V 1533

Parikh, NU 1710

Parisi, V 1809

Park, BH 455

Park, SR 1402

Park, YH 959

Parker, C 1361

Parkin, D 62

Parkin, DM 450

Parkinson, MC 499

Parks, RW 213

Parodi, F 1845

Paroush, Z 771

Parry, EM 891

Parry, JM 891

Pasetto, L 785

Passmore, SJ 22

Passoni, P 785

Patel, KK 1122

Patel, N 1446

Patel, PH 614

Paul, J 62

Paulissen, G 724

Paziewska, A 586

Pedersen, L 1339

Pedrazzoli, P 1016

Peeters, M 481

Peeters, PHM 299

Penault-Llorca, F 259

Pennington, CJ 569

Pennisi, G 1428

Pennucci, MC 1263

Pera, G 299

Peretz, T 681

Périé, S 1164

Perret, G-Y 1823

Perrier, H 69

Perrone, F 1233

Petridou, E 156

Petrillo, A 1809

Petroulakis, E 195

Pettersson, F 1853

Pezzella, F 1176

Pezzolo, A 1845

Pfeiffer, S 1672

Pharoah, PD 1921

Philippo, K 661

Philips, M 672

Philips, Z 1253

Pickles, MD 427, 1554

Piemonti, L 785

Pilotto, LS 1116

Pinedo, HM 1627

Pinel, M-C 1383

Pintér, T 1777

Pirie, L 1942

Pischon, T 299

Pistoia, V 1845

Pitchers, M 955

Plantade, A 1287

Platell, C 1116

Platt-Higgins, A 1057

Pless, M 1099

Polette, M 724

Pollice, AA 1144

Pollock, BH 161, 940

Polus, M 481

Polyzos, A 798

Pond, GR 1011, 1136

Ponder, BAJ 1921

Popa, J 578

Popat, S 18

Poston, GJ 982

Poulsen, AH 1339

Poveda, A 1797

Powell, MA 642

Powers, HJ 1942

Powles, T 51

Pozos, R 1176

Prall, F 976

Prendeville, J 1107

Price, A 1375

Primrose, JN 982

Priou, F 69

Proby, CM 1446

Prochilo, T 1263

Provencher, DM 436

Prudente, RY 654

Prus, D 681

Pühringer-Oppermann, F 203

Puisieux, A 13

Qian, W 806

Quan, L 686

Quesada Calvo, F 724

Quinn, M 1751

Quirós, JR 299

Rabitti, C 792

Rabkin, CS 548

Ramaioli, A 1516

Ramlau, R 1375

Ramsey, S 781

Rapiti, E 231

Ratel, D 1066

Ray, A 1765

Raymond, JS 1745

Redhead, DN 213

Redmond, M 1320

Reed, JE 1186

Reed, MWR 101

Reed, NS 62

Rees, M 982

Rees, MCP 1

Reeves, P 1186

Reitz, M 1785

Rembacken, BJ 311

Ren, F 436

Reni, M 785

Rensch-Boschert, K 578

Renshaw, L 1051

Ribatti, D 1845

Riboli, E 299

Ricci, S 473, 1164

Riede, U 128

Riemann, JF 1572

Rinaldi, S 299

Ring, AE 358

Riofrio, M 1610

Ris, H-B 1099

Risk, JM 561

Risuglia, N 1428

Ritter, G 681

Rivory, L 964

Robaszkiewicz, M 1214

Robboy, SJ 1734

Roberts, ISD 1949

Rocks, N 724

Rodenhuis, S 1226

Rodriguez, C 757

Rogers, SJ 631

Rogers, SN 561, 647

Rohan, TE 757

Rojo, F 1293

Rolland, F 1395

Romain, S 239

Romer, G 43

Ronnstrand, L 1180

Rosen, EM 407

Roskelley, C 1326

Ross, JA 731, 1663

Ross, PJ 1107

Ross, RJ 469

Ross, SR 548

Rossi, E 1233

Rossing, MA 1071

Rosso, S 743

Rosti, G 1016

Roth, AD 1099

Rothermundt, C 74

Rothman, KJ 142

Roy, D 1524

Roystacher, M 681

Royston, P 1785

Rozendaal, L 171

Rudant, J 763

Rudland, PS 1057

Ruggeri, EM 1789

Russell, C 1107

Russell, I 1253

Russo, F 1263

Rustin, GJS 62, 1231

Ruszkiewicz, A 593

Rutter, C 1071

Rykova, EY 1492

Ryoo, B-Y 959

Ryu, KW 1402

Saad, F 1906

Sabokbar, A 1496

Sadanaga, N 1894

Sadler, G 828

Saffroy, R 1180

Sagae, S 914

Saintigny, P 1164

Saisho, H 1575

Saito, H 1599

Saito, K 717

Saito, T 914

Saitoh, S 1803

Saka, H 1599

Sakata, R 737

Sakata, Y 1803

Salmon, A 308

Salonen, L 1697

Salud, A 969

Samali, A 398

Samonis, G 798

Sancho-Garnier, H 743

Santini, D 792

Sappino, AP 231

Sarbia, M 203

Sarp, S 231

Sasaguri, Y 710

Sasaki, A 1894

Sasaki, F 1510

Sasazuki, S 740

Sasieni, P 460

Sato, A 1586

Satoh, Y 1485

Saunders, PTK 1333

Sauter, G 128

Savage, P 51, 1217

Sawada, T 1510

Sawyer, TK 1710

Scagliotti, GV 1375

Scanlan, M 681

Schaap, GR 1837

Schäfer, C 1544

Schagen, FHE 1837

Schaller-Kranz, T 79

Scharpé, S 672

Schatzkin, A 757

Scheithauer, W 1122

Schernhammer, ES 928

Schiffman, M 1690

Schmeller, N 268

Schmid, RA 1099

Schmidt, M 1879

Schmiegel, W 1293

Schoemaker, MJ 1352

Schouten, LJ 165, 757

Schröder, FH 1093

Schrump, DS 1436

Schubert, H 231

Schubert, P 578

Schwartz, R 1144

Schwarz, S 1592

Schymura, MJ 1738

Scolyer, RA 904

Scoto, GM 1428

Scott, C 904

Scott, IS 1170

Scurry, JP 904

Seckl, MJ 51

Sehouli, J 1615

Seidel, A 1726

Seidel, P 1726

Seifalian, AM 1213

Seike, K 1816

Seimiya, H 341

Seitz, JF 69

Senan, S 625

Senn, H-J 524

Seshadri, T 1007

Seymour, JF 1007

Seymour, MT 1281

Shackney, SE 1144

Shah, E 828

Shahbazian, D 195

Shakespeare, WC 1710

Sham, JST 108

Shamimi-Noori, S 1436

Shamir, R 1537

Shankley, N 1107

Shanley, S 308

Shapiro, LH 1627

Sharma, R 964

Sharma, S 1029

Sharp, L 1253

Shaw, DE 1621

Shaw, E 1777

Shaw, EJ 1186

Shaw, RJ 561

Shearer, CJ 1568

Shepherd, FA 1452

Shepherd, N 1460

Sherman, ME 1690

Shetty, G 1460

Shibahara, T 698

Shibata, A 737

Shibata, K 552

Shida, T 1816

Shiiba, M 717

Shilling, V 828

Shimada, K 532

Shimajiri, S 710

Shimamura, T 1803

Shimizu, N 247, 599

Shimokata, K 1599

Shin, HJ 1407

Shinomura, Y 914

Shioiri, M 1816

Shionoya, H 854

Shipley, J 308

Shoji, T 1586

Short, D 51

Sibson, DR 1057

Sier, CFM 1035

Sierra, M 1797

Siersema, PD 1389

Silagy, C 1116

Silva, JM 161, 940

Simes, RJ 208

Simmonds, PC 982

Simon, R 128

Singh, KP 1524

Singh, S 85

Siu, LL 1136

Sjöström, M 1853

Skriver, MV 142

Skvortsova, TE 1492

Slimani, N 299

Smerage, JB 8

Smith, AN 904

Smith, CA 1144

Smith, D 1281

Smith, IE 358

Smith, P 806

Smith-Warner, SA 757

Snijders, PJF 171

So, T 896

Soda, H 1267

Sohn, SK 1407

Sonenberg, N 195

Song, H 1921

Song, SY 1678

Sonoda, T 914

Soo, KC 1546

Soon, Y 922

Sorbe, B 55

Sorensen, BS 1703

Sørensen, HT 142, 1339

Souglakos, J 798

Spaderna, S 1672

Spencer, A 1007

Sperl, dW 268

Spiegelman, D 757

Spielmann, M 1610

Spiliopoulos, A 1099

Spiro, SG 1763

Spisni, E 1300

Squire, JA 1452

St. James-Roberts, I 43

Stacker, SA 1355

Stahel, R 1099

Stahl, M 203

Staiger, W 578

Stanta, G 879

Starikov, AV 1492

Stassi, G 578

Stea, B 1777

Steffen, RP 1777

Steidler, A 578

Stein, R 828

Stephan, C 540

Stephenson, TP 891

Stern, C 1007

Steward, WP 1420

Stiller, CA 22

Stockler, MR 208

Stoehlmacher, J 281

Stone, N 1460

Storey, A 1446

Stratford, IJ 115

Stratton, MR 318

Streit, S 1879

Stricker, BHCh 752

Strieter, RM 1029

Strillacci, A 1300

Strohsnitter, WC 1734

Stuart, RC 637, 1568

Stueber, C 281

Stupp, R 1099

Südhoff, T 1293

Sudo, K 1575

Sugaya, M 896

Suggett, N 1898

Sugihara, K 1130

Sugimachi, K 1894

Sugimoto, T 1510

Sugio, K 896

Sugiyama, T 1369, 1586

Suh, JH 1777

Suita, S 1510

Summersgill, B 308

Summy, JM 1710

Sundar, SS 1650

Sutherland, RL 904

Suzuki, H 737, 914

Suzuki, K 1874

Suzuki, M 1369

Suzuki, R 1599

Suzuoki, M 275

Sweetman, J 499, 507

Swerdlow, AJ 1352

Symonds, R 62

Syrigos, K 798

Syrigos, KN 631

Szakmany, T 647

Szczesna, A 1375

Szegezdi, E 398

Taamma, A 1610

Tabone, S 1180

Taioli, E 1533

Tajiri, T 1510

Takabatake, D 247

Takagi, S 1580

Takahashi, H 247

Takahashi, O 1586

Takaku, T 599

Takano, M 1369

Takano, S 1816

Takano, T 1586

Takatani, H 1267

Takeuchi, H 1874

Takeuchi, S 1369

Takimoto, M 311

Takiuchi, H 1803

Tamakoshi, A 737

Tamkovich, SN 1492

Tan, EH 1546

Tanaka, F 1894

Tanaka, T 1586

Tanay, M 631

Taniere, P 922

Taniguchi, H 1599

Tanimoto, A 710

Tannock, IF 863, 1011

Tanzawa, H 698, 717

Tao, L 1164

Tao, Y 108

Tase, T 1586

Tatangelo, F 1809

Tattersall, MHN 208

Tazawa, H 854

Teague, J 318

Téhard, B 299

Tenen, DG 1918

Teng, NN 642

Teodoridis, JM 1087

Terasawa, K 914

Ternullo, MP 578

Terracciano, L 128

Terzoli, E 1789

Thar, TT 287

Theis, B 1107

Théobald, S 1041

Theodore, C 1395

Théou-Anton, N 1180

Thomas, AL 1420

Thomas, G 1472

Thomson, BNJ 213

Thörn, M 1478

Thürlimann, B 524

Thykjaer, T 1703

Tibaldi, C 1263

Tiedemann, K 1007

Tijssen, CC 752

Tilanus, HW 1389

Tillner, J 1293

Tilney, C 43

Timoshenko, AV 1154

Tirmarche, M 1342

Tisdale, MJ 731

Titus-Ernstoff, L 1734

Tokino, T 914

Tomasi, V 1300

Tonelli, D 69

Tonin, PN 436

Tonini, G 792

Topham, C 1281

Tornesello, ML 446

Tornillo, L 128

Torp Madsen, HH 218

Tortora, G 1604

Tötsch, M 1099

Touboul, E 473

Tourani, J-M 1383

Townsley, CA 1136

Toyota, M 914

Tozer, DJ 427, 1554

Tran, TCK 1389

Tranah, GJ 928

Trevino, JG 1710

Trevisan, G 879

Trichopoulos, D 156, 299

Trichopoulou, A 299

Trodella, L 792

Troiani, T 1604

Troisi, R 1734

Tronko, N 1472

Tsao, M 1136

Tsao, M-S 1452

Tseng, C-P 870

Tsousis, S 798

Tsuchiya, E 1485

Tsuda, H 1369, 1369

Tsugane, S 740

Tsukuda, K 247

Tsurutani, J 1267

Tsutsui, S 1874

Tumino, R 299

Turnbull, LW 427, 1554

Turner, L 492

Turpin, F 1610

Twelves, C 1122

Tynes, T 1352

Uchiumi, T 710

Ueki, M 1369

Ullrich, A 1879

ünal, C 1293

Unger, K 1472

Uramoto, H 896

Utting, JF 1420

Uzan, S 473

Uzawa, K 698, 717

Uzzan, B 1823

Vacca, A 1845

Valsesia-Wittmann, S 13

Vamvakas, L 798

van Beers, EH 333

van Berkel, MPA 1627

van Beusechem, VW 1837

van Bochove, A 625

van Dam, P 672, 1643

van de Velde, CJH 1035

van de Water, B 661

van Dekken, H 1389

van den Brandt, PA 165

Van den Eynden, GG 1643

Van der Auwera, I 1643

van der Gaast, A 1389

van Duijn, CM 752

van Duijn, W 1035

van Eijk, R 661

van Gils, CH 299

van Kemenade, FJ 171

van Krieken, JHJM 1035

Van Laere, SJ 1643

Van Laethem, JL 481

Van Marck, E 672

Van Marck, EA 1643

van Meerbeeck, JP 625

van Meerten, E 1389

van Tinteren, H 625

van Wezel, T 661

Van Zandwijk, N 1375

Vanden Bergh, P 904

Vannucchi, AM 1637

Vardy, J 1011

Vasei, M 128

Vasey, P 55

Vasey, PA 62

Vaughan, ED 561, 647

Vaughan, N 1942

Veerakumarasivam, A 569

Verhoef, S 333

Verkooijen, HM 231

Vermeulen, PB 672, 1643

Verspaget, HW 1035

Vias, M 1326

Vicent, J-M 969

Vincenzi, B 792

Vineis, P 299

Vlassov, VV 1492

Vlastos, G 231

Volant, A 1214

von Briel, C 1099

von der Maase, H 218

von Minckwitz, G 1615

von Moos, R 524

von Weyhern, C 203

Voss, S 1759

Vowler, SL 1170

Vyas, J 1446

Waddell, ID 1663

Waddell, KM 446

Wagner, GF 1154

Wæhre, H 820

Wakelam, MJO 1898

Walch, A 1472

Walker, C 1186

Walker, LG 1253

Walker, M 1107

Wallard, MJ 569

Wallin, K-L 1045, 1913

Wallis, JC 22

Walsh, G 358

Walters, SJ 492

Wanders, A 1478

Wang, HJ 108

Wang, Y 686

Ward, DG 287, 1898

Warnke, P 1186

Warrack, R 922

Warren, A 569

Washio, K 247

Wasylyk, B 1041

Watanabe, G 593

Watanabe, T 293

Watson, M 43, 499, 507

Wattchow, DA 1116

Waxman, J 346

Webb, A 806

Webb, T 318

Weber, D 1293

Weder, W 1029, 1099

Wee, J 1546

Wei, W 287, 1898

Weichert, W 540

Weiner, L 1621

Weinhäusl, A 323

Weinlich, G 835

Weinreb, DB 1948

Weirich, G 203

Weisberg, E 1765

Weiss, C 578

Weiss, NS 1071

Weiss, R 1504

Welch, R 55

Weller, D 1272

Weller, DP 1116

Weller, I 1504

Welsh, FKS 213

Wen, JM 108

Went, P 128

Wernli, M 1099

West, CML 115

West, J 1751

Westermayer, S 1718

Whitaker, NJ 339

White, B 189

White, S 1051

Whitehead, S 828

Whynes, D 1253

Wibom, C 1853

Wigmore, SJ 213, 731, 1663

Wiklund, F 1913

Wild, SH 1079

Wilkinson, PM 55, 947

Willeke, F 578

Willett, WC 928

Williams, S 372

Wilson, C 227

Wilson, GD 121

Winqvist, O 1478

Winslet, MC 1213

Winstanley, J 828

Winter, DC 1320

Winzer, K-J 540

Wion, D 1066

Wisløff, T 1559

Witlox, MA 1837

Wittmer, C 281

Wolk, A 757

Woll, PJ 1936

Wolschke, C 281

Wood, H 1751

Woolley, CM 1253

Wooster, R 318

Wright, DJ 1663

Wright, J 469

Wuisman, PIJM 1837

Wuyts, H 672

Xie, D 108

Xu, D 1658

Xu, N 686

Yaegashi, N 1369, 1586

Yamada, H 1130, 1586

Yamaguchi, K 1803

Yamaguchi, T 1575

Yamamoto, E 552

Yamamoto, K 1510

Yamamoto, M 1599

Yamamoto, T 311

Yamashita, T 1130

Yang, S-C 1029

Yang, SH 959

Yang, W 1213

Yasuda, K 1874

Yasumoto, K 896

Yau, T 18

Yee, D 465

Yen, W-C 654

Yeow, W-S 1436

Yokoe, H 698, 717

Yokoyama, Y 1586

Yoshikawa, N 311

Yoshikawa, Y 1894

Yoshimura, T 737

Young, A 51

Yovine, A 1610

Yu, MS 1402

Yu, W 1407

Yuana, Y 1627

Yubero, A 969

Zamagni, C 1016

Zanetti, R 743

Zdeb, MS 1738

Zelek, L 1610

Zelger, B 835

Zeng, YX 108

Zerbi, A 785

Zerilli, M 578

Zetterberg, A 1045

Zhang, G 686

Zhang, H 1650

Zhang, SM 757

Zhang, Y 599

Zhao, S 1658

Zheng, BY 1913

Zhou, C 686

Zhou, X 686

Zhu, C-Q 1452

Zhu, H 686

Zhu, YM 1936

Ziauddin, MF 1436

Zielinski, GD 171

Zilis, D 299

Zimmerman, RME 661

Ziras, N 798

Zitzelsberger, H 1472

Zohar, S 609

zur Hausen, A 578

Zurnadzhy, L 1472

